# Dyslipidemia induced by antipsychotics: di”erences between schizophrenia and bipolar disorder

**DOI:** 10.1192/j.eurpsy.2024.504

**Published:** 2024-08-27

**Authors:** A. Tahirovic, M. Zuko, I. Lokmic Pekic, M. Arnautovic Tahirovic

**Affiliations:** ^1^Psychiatric clinic, Clinical centre university of Sarajevo; ^2^Psychiatric hospital of Canton Sarajevo, Sarajevo, Bosnia and Herzegovina

## Abstract

**Introduction:**

The introduction of antipsychotics, especialy of newer generation, greatly a”ects the e”ectiveness of the psychiatric treatment of patients with schizophrenia (SCH) and bipolar disorder (BP). Patients su”ering from SCH and BP often have metabolic syndrome (MetSy), as a result of taking antipsychotic therapy, especially in patients with abdominal obesity, there is an atherogenic fat profile that carries a high risk for the development of dyslipidemia.

**Objectives:**

To investigate frequency and di”erences of somatic diseases in patients with SCH and BD depending on the presence of MetSy.

**Methods:**

This five-year prospective study was conducted in the Psychiatric Hospital of Canton Sarajevo. We followed 135 patients with SCH and 135 patients with BD, aged 30 to 69 years, who were treated with antipsychotics for five years.

**Results:**

Dyslipidemia was significantly more common in SCH patients (73.3%), compared to BD (54.1%) and was dominantly presented in women (61.4%). The frequency of dyslipidemia increased with the age of the patient. Associated risk factors in patients with SCH diagnosed with dyslipidemia were 73.5% smokers, 78.7% hypertensive patients, 69.7% patients with elevated BMI and 83.0% with elevated blood glucose values, while slightly lower values were recorded patients with BP. 97.8% of patients with dyslipidemia had elevated CRP.

**Conclusions:**

There are significant di”erences in dyslipidemia in patients su”ering from SCH and BP. Adequate knowledge of the antipsychotic drugs is required in order to provide adequate psychiatric treatment, regarding minimalising adverse e”ects of antipsychotics will be reduced to a minimum. It is important to recognize high-risk patients and educate them about preventive measures.

**Disclosure of Interest:**

None Declared

